# Case Report: A case of multisystem inflammatory syndrome in an 11-year-old female after COVID-19 inactivated vaccine

**DOI:** 10.3389/fped.2023.1068301

**Published:** 2023-02-14

**Authors:** Saboor Saeed, Jianqing Cao, Jinjiao Xu, Yi Zhang, Xuyang Zheng, Liya Jiang, Chunming Jiang, Xinjuan Zhang

**Affiliations:** ^1^ Department of Pediatrics, International Education College of Zhejiang Chinese Medical University, Hangzhou, Zhejiang, China; ^2^ Department of Pediatrics, Affiliated Hangzhou First People's Hospital, Zhejiang University School of Medicine, Hangzhou, Zhejiang, China; ^3^ Department of Pediatrics, The Fourth School of Clinical Medicine, Zhejiang Chinese Medical University, Hangzhou, Zhejiang, China

**Keywords:** SARS-CoV2, covid-19 inactivated vaccine, multisystem inflammatory syndrome, methylprednisolone, coronavirus disease 2019 (COVID-19)

## Abstract

**Background:**

Multisystem inflammatory syndrome in children (MIS-C), also known as pediatric inflammatory, multisystem syndrome temporally associated with SARS-CoV-2, is a rare but serious complication of SARS-CoV-2 infection in children that typically occurs 2–6 weeks after SARS-CoV-2 infection. The pathophysiology of MIS-C is unknown. MIS-C, first recognized in April 2020, is characterized by fever, systemic inflammation, and multi-system organ involvement. Post-vaccination adverse effects have increased with COVID-19 vaccinations, and MIS linked to immunization with COVID-19 vaccines has also been observed.

**Case Report:**

An 11-year-old Chinese girl presented with a high-grade fever, rash, and dry cough for 2 days. She had her 2nd SARS-CoV-2 inactivated vaccination dose five days before hospital admission. On day 3 & 4, she experienced bilateral conjunctivitis, hypotension (66/47 mmHg), and a high CRP level. She was diagnosed with MIS-C. The patient's condition deteriorated rapidly, necessitating intensive care unit admission. The patient's symptoms improved after intravenous immunoglobulin, methylprednisolone, and oral aspirin therapy. She was discharged from the hospital after 16 days as her general condition, and laboratory biomarkers returned to normal.

**Conclusion:**

Inactivated Covid-19 vaccination might trigger MIS-C. Further research is needed to evaluate whether a correlation exists between COVID-19 vaccination and MIS-C development.

## Introduction

Global immunization against SARS-COV-2 (severe acute respiratory syndrome-2-coronavirus), which causes coronavirus disease-2019 (covid-19), commenced in December 2020 (1). Simultaneously, since vaccination began, the prevalence of COVID-19 cases and mortality have decreased significantly. Vaccines have different mechanisms based on nucleic acids (RNA and DNA), protein subunits, and inactivated viruses (2). Therefore, all kinds of vaccines are always potentially associated with various adverse effects. And the term “Adverse Events Following Immunization (AEFI)” has come to mean “any adverse medical incident that occurs post-vaccination and is not necessarily linked to vaccination” (3, 4).

A rare but severe complication of SARS-COV-2 infection in pediatric practice is MIS-C, also known as pediatric inflammatory, multi-system syndrome, temporally linked with SARS-COV-2. It typically manifests 2–6 weeks after the onset of the SARS-CoV infection. MIS-C was first described in April 2020. It is marked by fever, systemic inflammation, and multiple organ involvement (5). MIS-C post-COVID-19 vaccination is challenging to diagnose since it lacks diagnostic biomarkers. Its manifestations may resemble other diseases, such as Kawasaki disease, toxic shock syndrome, and acute COVID-19 infection (6). The pathophysiology of this syndrome remains unclear. We present the case of an 11-year-old girl diagnosed with MIS-C five days after receiving her second dose of the inactivated Covid-19 Vero cell vaccination. Treatment with intravenous immunoglobulin (IVIG) and methylprednisolone was effective. Cases of MIS-C have been reported after the COVID-19 vaccination (7). To the best of our knowledge, this is the first and youngest case of MIS-C associated with 2 doses of inactivated COVID-19 vaccination so far. This case report is essential because it draws attention to this syndrome and provides a model for clinicians working with children and adults.

## Case presentation

A healthy 11-year-old female presented to the pediatric ward of our hospital 5 days after receiving her second SARS-COV-2 inactivated vaccine (left deltoid) with 3 days of high-grade fever, dry cough, and rash for 2 days. She received her first dose of inactivated SARS-CoV-2 vaccine four weeks before her hospital visit. She denied recent viral infections, previous COVID-19 infection, known contact with patients infected with SARS-COv-2, cutaneous injuries, and international travel., The only medication taken recently was azithromycin, prescribed at the local hospital two days after the onset of the disease. The child's fever and rash symptoms started before receiving azithromycin. Therefore, a possible adverse drug reaction was not considered. During a physical examination, her body temperature was 39.2 °C, heart rate was 140/min, respiration rate was 26/min, blood pressure was 101/67 mmHg, and oxygen saturation in the ambient atmosphere was 98%. A generalized erythematous pruritic maculopapular rash, cracked lips, strawberry tongue, normal cardiac and lung auscultation; soft abdomen, liver and spleen were not palpable under the ribs; swollen limbs without skin peeling.

The initial laboratory examination upon admission showed a peripheral white blood cell (WBC) count of 5.3*10^9^/l, with neutrophils accounting for 82.2% and lymphocytes for 5.9%; C-Reactive protein (CRP) levels were elevated at 89.8 mg/dl. Nasopharyngeal SARS-CoV-2 RT-PCR was negative; SARS-CoV-2 spike antibody testing was positive; Troponin level and echocardiogram were normal.

**Figure 1:**
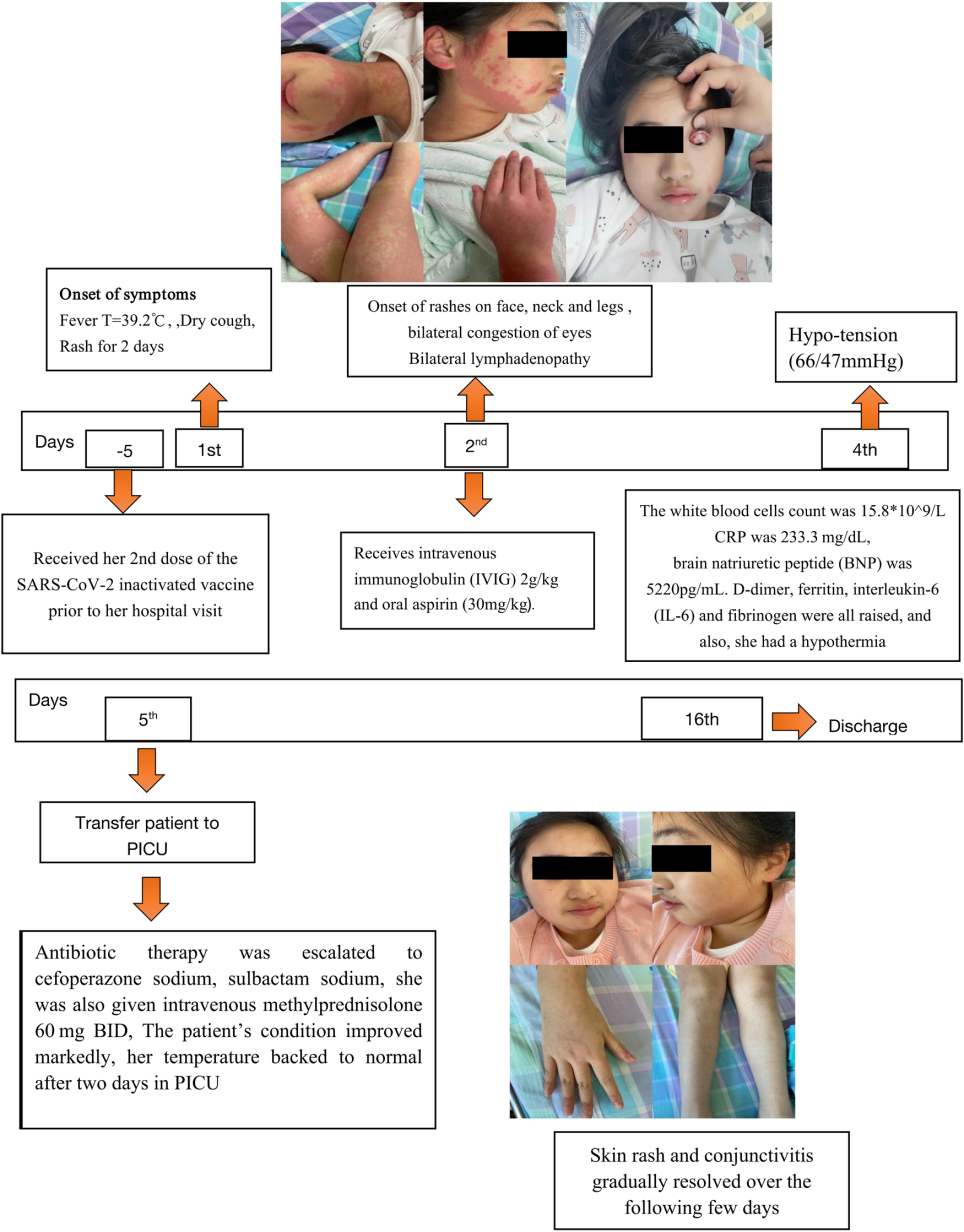
Timeline showing the clinical presentation and follow-up of the patient with multisystem inflammatory syndrome after COVID-19 vaccination. BNP, brain natriuretic peptide; CRP, C-reactive protein; IL-6, interleukin-6; PICU pediatric intensive care unit.

On the second day of admission, she experienced bilateral conjunctivitis, enlarged lymph nodes in the neck, edema of both hands and feet. (shown in Figure 1), and abdomen discomfort with nausea. Overall, the patient's symptoms are similar to those of Kawasaki disease on the third day of hospitalization. We initially suggest diagnosed of Kawasaki disease and the patient received 2 g/kg of intravenous immunoglobulin (IVIG) and 30 mg/kg of oral aspirin. A day later, she was noted to have hypotension (66/47 mmHg), tachycardia, hyperlactatemia, metabolic acidosis, and electrolyte imbalance. In addition, Laboratory tests show her inflammatory markers were increased (Table 1). The white blood cells count was 15.8*10^9^/l, with decreased lymphocyte counts. The CRP level was elevated at 233.3 mg/dl, the troponin (TNI) level was 0.44 ug/l, and the brain natriuretic peptide (BNP) level was 5220 pg/ml. In addition, she had elevated levels of D-dimer, ferritin, interleukin-6 (IL-6), fibrinogen, and hypoproteinemia. An ECG revealed sinus tachycardia and a slight T wave alteration. A transthoracic echocardiogram revealed no abnormality. She was admitted to the pediatric intensive care unit (PICU) for suspected multisystem inflammatory syndrome in children (MIS-C). Potential diagnoses include septic shock, multisystem inflammatory syndrome post COVID-19 vaccine (MIS-V), Mycoplasma pneumonia-induced rash and mucositis/Stevens-Johnson Syndrome (MIRM/SJS), post-viral peri myocarditis, and Macrophage activation syndrome.

**Table 1:** Laboratory indexes.

Test projects	Day 1	Day 4	Day 5	Day 6	Day 7	Day 9	Day 11	Day 13	Day 16
WBCs (10^9^/l) (3.5–9.5)	5.3	12.8	15.8	21.3	16.7	18.4	19.4	21.3	17.3
Neutrophil (%) (40–75)	82.2	85.9	94.5	93.3	88.2	82.7	85.3	83	70
lymphocytes (%) (20–50)	5.9	5.1	4.3	5.4	9.2	12.5	12.3	14	25.4
Hemoglobin (g/l) (115–150)	115	109	109	124	116	119	117	126	130
Platelets (10^6^/l) (125–350)	153	164	210	213	257	366	335	379	402
CRP (mg/dl) (<1.00)	89.8	233.3	211.3	187.8	100.9	21	22.5	8.6	2.9
PCT (ng/ml) (0.0–0.5)	0.68		3.87	1.84	1.14	0.4		0.25	
ESR (mm/H) (0–15)	31								14
D-dimer (mg/l) (0–500)	1620		910					810	
Ferritin (ng/ml) (4.63–204)			485.42	540.11		368.97			
TNI (ug/l) (0.00–0.11)	<0.01		0.44	0.25	0.2	0.13		0.12	0.03
BNP (pg/ml) (0–400)	160		5220	4140	3290	1410		844	129
CK (U/l) (24–195)			51	26	18	16		39	33
CKMB (U/l) (1–25)			19	11	13	12		19	22
albumin (g/l) (40–55)	33.9		23.4		23.5	25.8		26.1	32
IL-6 (pg/ml) (0–20)		62.11			162.82		2.41		
Fibrinogen (g/l) (2–4)	4.66		5.6						
Blood culture				Negative			Negative		
Urine culture			Negative						
Bacterial throat cultures			Negative						
SARS-CoV-2 RT-PCR	Negative								
SARS-CoV-2 antibody IgG	Positive								
Respiratory infection pathogens IgM		Negative							

WBC, white blood cells; CRP, C-reactive protein; PCT, procalcitonin; ESR, erythrocyte sedimentation rate; TNI, troponin; BNP, brain natriuretic peptide; C.K., creatine kinase; CKMB, creatine kinase myocardial band; IL-6, interleukin-6.

The symptoms of shock improved after fluid resuscitation, intravenous albumin improved hypoproteinemia, and antibiotic therapy was escalated to cefoperazone sodium and sulbactam sodium. Furthermore, she received intravenous methylprednisolone 60 mg bid. The patient's symptoms significantly improved. After two days of stable vital signs in the PICU, her temperature returned to normal, and the most inflammatory markers returned to normal levels. After 5 days of treatment with intravenous methylprednisolone, the levels of troponin and BNP dropped to 0.13 ug/l and 1,440pg/ml, respectively. Then switched to methylprednisolone orally. All tests for pathogens were negative. Therefore, we ruled out the possibility of other infectious disorders and terminated antibiotic treatment.

After being hospitalized for 16 days, the laboratory indicators reverted to normal, the child was discharged from the hospital and allowed to go home. After discharge, methylprednisolone was discontinued two weeks later. At 8 weeks post-discharge, she remained healthy, and aspirin was discontinued.

## Discussion

We report a previously healthy child diagnosed with MIS-C, 5 days after receiving 2 doses of inactivated COVID-19 vaccine. The patient's clinical presentation resembled Kawasaki disease (KD), and she was treated with intravenous immunoglobulin, methylprednisolone, and oral aspirin. According to the Brighton criteria, MIS-V should occur within 12 weeks of SARS-CoV-2 immunization. Therefore, even though it is unclear that the vaccine directly caused MIS-C, but still we cannot rule out the possibility of a relationship between them.

Recent studies and data showed that covid-19 vaccines are safe and well-tolerated. However, there are still some concerns regarding its side effects. Some mild side effects of covid-19 vaccines include low-grade fever, fatigue, pain at the injection site, and pain in the musculoskeletal. Adverse reactions often begin within three days of vaccination and resolve within a few days (8, 9). Some recent studies highlight that severe side effects of covid-19 vaccines have been reported, such as myocarditis, especially in male adolescents. MIS-C post-vaccination is a rare condition, in pediatric patients. Based on the identification of MIS-C, a similar disorder in adults is known as (MIS-A). Although the pathogenesis of MIS is unclear, both MIS-C and MIS-A show post-infectious manifestations of COVID-19. The Brighton Collaboration network identified both disorders as post-vaccination side effects of COVID-19 vaccinations (10). Several adult cases describe the incidence of MIS-A post-COVID-19 vaccination, yet it is unknown whether MIS-C/A may result after COVID-19 vaccination (11).

After the widespread use of COVID-19 vaccines, significant difficulty in serologic discrimination has emerged: how to distinguish between an antibody response created by the vaccine and one caused by SARS-CoV-2 infection. It is known that the vaccine induces a reactive reaction on anti-SARS-CoV-2 spike antibodies, However, distinguishing between prior infection and prior vaccination may be difficult, as SARS infection may also cause these antibodies. On the other hand, vaccination does not result in a reactive test result for the anti-SARS-CoV-2 nucleocapsid antibody produced during and after an infection. For this reason, it has been proposed that measurements of anti-spike and anti-nucleocapsid-based serology be performed to differentiate between reactions induced by spike-protein-based immunization and those caused by spontaneous infection (12).

As the mechanism of inactivated vaccines differs from that of mRNA vaccines, there is no evidence that anti-spike and antinucleocapsid-based serology can be used to diagnose MIS-V post-inactivated vaccination. In China, the inactivated covid-19 vaccination was initially administered to children ages 12–17 in July 2021, while the same year, in October, the age range was expanded to cover children ages 3–11. Consequently, the number of people experiencing covid-19 side effects increases in tandem with the number of vaccinated people. In this study, we present the case of an 11-year-old female patient who developed MIS-C despite showing no clinical signs of covid-19 infection after receiving two doses of the inactivated covid-19 vaccination. We did not conduct serological testing for anti-spike or anti-nucleocapsid antibodies. According to the Brighton criteria. SARS-CoV-2 vaccination results in MIS-V within 12 weeks, there is a substantial temporal correlation between the two events, even though it cannot be proven with complete certainty that the vaccination caused the MIS. Therefore, one cannot totally rule out a connection between MIS-C and the inactivated covid-19 vaccine. Due to the temporal association, this AEFI was classed as uncertain with a causal relationship to vaccination; however, further evidence that the vaccine caused the event is required (13).

All currently reported cases are associated with receiving one or more doses of mRNA COVID-19 vaccination before disease onset; no cases of MIS have been reported because of inactivated COVID-19 vaccination. It is necessary to continue and carry out extensive AEFI awareness as it will increase our understanding of various clinical symptoms related to multiple COVID-19 vaccines. MIS is a rare and severe complication of COVID-19 infection, and there have been few reports of similar clinical syndromes occurring only after vaccination. To the best of our knowledge, our patient is the first and youngest reported case of MIS-C associated with two doses of inactivated COVID-19 vaccination. Studies from Turkey, Saudi Arabia, and Canada reported a minimum age of 12 years for COVID-19 vaccine-associated MIS. Which may be related to the fact that some countries did not fully promote COVID-19 vaccination among children aged 3–11 years (14–16). Continued monitoring for MIS-C syndrome post-COVID-19 vaccination is essential, especially considering that pediatric COVID-19 vaccination is approved for younger children who are at high risk for MIS-C syndrome after SARS-CoV-2 infection (17). To ensure vaccination safety, doctors should closely monitor vaccinated people for symptoms of severe or unexpected adverse effects. In addition, further monitoring, and precise analysis of adverse reactions to the COVID-19 vaccine are also required, particularly in cases involving MIS.

## Conclusion

In conclusion our case suggests that inactivated COVID-19 vaccination could result in MIS-C. However, more research is needed to identify if there is a link between COVID-19 vaccination and the development of MIS-C. Also, MIS-C requires exploratory research and investigation. Therefore, children with COVID-19-post vaccination should be given special attention.

## Data Availability

The original contributions presented in the study are included in the article/Supplementary Material, further inquiries can be directed to the corresponding author/s.
